# Combined Application of Biochar and Plant Growth-Promoting Rhizobacteria Improves Heavy Metal and Drought Stress Tolerance in *Zea mays*

**DOI:** 10.3390/plants13081143

**Published:** 2024-04-19

**Authors:** Vadivel Anbuganesan, Ramasamy Vishnupradeep, L. Benedict Bruno, Krishnan Sharmila, Helena Freitas, Mani Rajkumar

**Affiliations:** 1 Department of Environmental Sciences, Bharathiar University, Coimbatore 641046, India; anbuganesh32@gmail.com (V.A.); vishnupradeep878@gmail.com (R.V.); brunobtech@gmail.com (L.B.B.); sharmilakrishnan98@gmail.com (K.S.); 2 Centre for Functional Ecology—Science for People & the Planet, Department of Life Sciences, University of Coimbra, 3000-456 Coimbra, Portugal; hfreitas@uc.pt

**Keywords:** biochar, rhizobacteria, drought stress, plant growth, soil enzymes, antioxidants, heavy metals

## Abstract

Plants are often exposed to multiple stresses, including heavy metals (HM) and drought, which limit the plant growth and productivity. Though biochar or plant growth-promoting rhizobacteria (PGPR) have been widely used for alleviating HM or drought stress in plants, the study of the effects of combined treatment with biochar and PGPR under simultaneous HM and drought stress is limited. This study investigated individual and combined effects of groundnut shell biochar (GS-BC) and PGPR *Bacillus pseudomycoides* strain ARN7 on *Zea mays* growth, physiology, and HM accumulation, along with their impact on soil enzymes under HM (Ni and Zn), drought, or HM+drought stress. It was observed that even under HM+drought stress, *Z. mays* growth, total chlorophyll, proteins, phenolics, and relative water contents were increased in response to combined GS-BC and ARN7 treatment. Furthermore, the combined treatment positively influenced plant superoxide dismutase, ascorbate peroxidase, and catalase activities, while reducing electrolyte leakage and phenolics, malondialdehyde, and proline under HM, drought, or HM+drought stress. Interestingly, the combined GS-BC and ARN7 treatment decreased HM accumulation and the bioaccumulation factor in *Z. mays*, highlighting that the combined treatment is suitable for improving HM phytostabilization. Additionally, GS-BC increased soil enzymatic activities and ARN7 colonization irrespective of HM and drought stress. As far as we know, this study is the first to illustrate that combined biochar and PGPR treatment could lessen the adverse effects of both HM and drought, suggesting that such treatment can be used in water-deficient HM-contaminated areas to improve plant growth and reduce HM accumulation in plants.

## 1. Introduction

Heavy metal (HM) pollution is one of the major environmental stresses that adversely affects plant growth and physiological process by altering protein structure, redox balance, membrane structure, intracellular enzymes, hormonal balance, etc., which in turn reduces crop productivity [1,2]. In addition to HM toxicity, the other environmental stresses, particularly drought, in HM-contaminated soils not only affect plant growth, including important physiological and biochemical processes such as water potential, stomatal closure, nutrient metabolism, respiration, translocation, and photosynthesis [3], but also aggravates HM stress by increasing HM bioavailability in soils and thus its uptake by plants [4], making the plants more susceptible to HM stress.

The amendment of soil with biochar improves the fertility, quality, and enzyme activity of soil which play a pivotal role in improving the tolerance of plants to various abiotic stresses including HMs [5]. The oxygenated functional groups of biochar induce HM immobilization through adsorption, precipitation, ion exchange, electrostatic interaction, etc., which potentially protects the plants and microbes from the toxic effects of HM [6]. Under HM stress, the application of biochar derived from various agriculture biomass, including banana pith, cotton straw, rice straw, etc., improves plant growth, nutrient uptake, physiology, and HM stress tolerance by improving antioxidant enzyme activities such as superoxide dismutase (SOD), ascorbate peroxidase (APX), catalase (CAT), etc. [7,8,9]. An increment in the activity of extracellular enzymes including β-glucosidase, which is involved in soil nutrient cycling, has also been reported with the addition of biochar in HM-contaminated soils [10,11]. Similarly, recent studies have also found that biochar amendment is an effective drought mitigation strategy as it improves soil’s water holding capacity and water retention and thus plant water-use efficiency by holding water in the pores and slowly releasing it under drought condition [12,13]. An increase in plant growth, chlorophyll contents, nutrient uptake, stomatal conductance, transpiration rate, photosynthesis, etc., was also reported under drought stress as the results of biochar amendment [12,13]. In addition, biochar, with volatile organic compounds, free radicals, large surface area, developed porous structure and various nutrients, including C, N, etc., can potentially increase microbial growth and activity in marginal soils by providing a protective habitat and a source of nutrients [14,15].

Recently, bioaugmentation with plant growth-promoting rhizobacteria (PGPR) has also gained great attention as an effective strategy to mitigate various abiotic stresses in plants and improve crop productivity [16,17,18]. PGPR with the potential to tolerate abiotic stresses including HM, drought, etc., can colonize the plant rhizosphere and exhibit beneficial effects on plants by synthesizing various plant growth-promoting (PGP) substances such as 1-aminocyclopropane-1-carboxylate deaminase, siderophores, indole-3-acetic acid (IAA), exopolysaccharides, antimicrobial metabolites, N fixation, and solubilizing P, K, Zn, etc. [7,18]. Moreover, such microbes can effectively increase HM and drought stress tolerance in plants by triggering the metabolic pathways of the plant, stimulating antioxidant production, modulating the expression of specific stress-responsive genes, etc. [19,20]. In a recent study, Vishnupradeep et al. [17] inoculated HM- and drought-tolerant *Providencia* sp. and *Proteus mirabilis* in the roots of *Zea mays* plants and witnessed both bacteria-accelerated plant growth under HM and drought stress by increasing photosynthetic efficiency, phenolics, and relative water content, and reducing proline content, the malondialdehyde (MDA) level, and SOD activity.

Considering the significance of biochar in improving physicochemical and biological properties of soils [5] and plant growth and physiological attributes under various abiotic stresses [8,12] and the beneficial effects of PGPR on plant growth under HM and/or drought stresses [17,18], the combined application of biochar and PGPR were examined in this study. It was hypothesized that the biochar amendment and (thus) improved survival and activity of inoculated PGPR may protect the plants from HM and drought stresses by modulating plant physiology and HM uptake. The objectives of the current study were as follows: (1) to prepare and characterize groundnut shell biochar (GS-BC), (2) to isolate and characterize a HM- and drought-tolerant PGPR, and (3) to investigate the individual and combined effects of GS-BC and PGPR on *Z. mays* growth, physiology and HM accumulation, along with their impact on soil enzymes under HM (Ni and Zn), drought, or HM+drought stress conditions.

## 2. Results and Discussion

### 2.1. Characterization of the Groundnut Shell Biochar (GS-BC)

**Figure 1:**
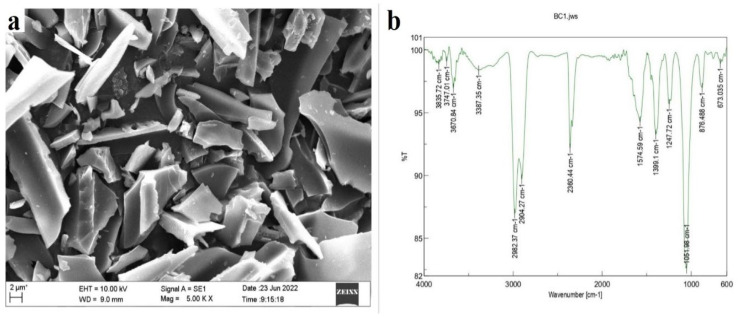
Surface morphology and functional groups of GS-BC. (**a**) SEM image and (**b**) FT-IR spectra of GS-BC.

In order to characterize the GS-BC, physical, chemical and elemental analyses were carried out (Table S1). The prepared GS-BC had a slight alkaline pH (7.7) and a high cation exchange capacity (CEC) of 7.9 cmol kg^−1^. High pH and CEC are an important characteristic feature of biochar, which can bind and retain the nutrients from leaching by reducing the acidification and thus enhance nutrient phytoavailability [21]. Further, the elemental and nutrient analysis revealed that the GS-BC had a high amount of carbon (60%), phosphorous, and potassium. The SEM analyses of the GS-BC revealed that it had a relatively smooth surface with few channels and pores (Figure 1a). Notably, the surface area of the GS-BC was found to be relatively large (3.5 m^2^ g^−1^), demonstrating that GC-BS has the potential to decrease the mobility and phytoavailability of HM through adsorption [22,23]. Furthermore, the functional groups of the GS-BC were identified through FTIR spectra (Figure 1b), and the vibrations were assigned as C-Br (halo compound; ~673.035 cm^−1^), C=C (alkene; ~876.488 cm^−1^), CO-O-CO (anhydride; ~1051.98 cm^−1^), C-N (amine; ~1247.72 cm^−1^), O-H (carboxylic acid; ~1399.1 cm^−1^), C=C (cyclic alkene; ~1574.59 cm^−1^), P-H (phosphine; ~2360.44 cm^−1^), N-H (amine salt; ~2904.27 cm^−1^), O-H (carboxylic acid; ~2982.37 cm^−1^), N-H (aliphatic primary amine; ~3387.35 cm^−1^), and O-H (alcohol; ~3670.84 cm^−1^) [24]. The presence of these functional groups also suggests that the GS-BC has great potential for the removal of various pollutants including HMs [6,25], since these functional groups serve as chelating agents, stabilizers, and/or ligands, which are involved in HM complexation, solubilization, stabilization, extraction, sequestration, immobilization, and ion exchange reactions [25].

### 2.2. Characterization of Strain ARN7

The HM tolerance and PGP features of the strain ARN7 under HM+drought condition were assessed (Table S2). The strain ARN7 exhibits tolerance to several HMs (Ni, Cu, Zn, Cd, and Cr); produces IAA, siderophores (hydroxamate), and exopolysaccharides; and solubilizes phosphorus in HM-infused medium under PEG-induced drought condition. These PGP features play an important role in plant–microbe interactions and can be harnessed to improve plant productivity and resilience to stress through various mechanisms including iron acquisition, nutrient absorption, enhancing soil structure, water retention, and nutrient uptake [17,26]. The plant growth-promotion ability of ARN7 was further confirmed with in vitro studies, where ARN7 increased the root length, shoot length, and fresh weight of the plant. The strain ARN7 was identified at the species level as *Bacillus pseudomycoides* via the 16SrRNA gene sequence and evolutionary relatedness of ARN7 with other bacterial strains, as constructed with the neighbor joining method and represented in a phylogenetic tree (Figure S1). The obtained sequence has been deposited in the NCBI GenBank under accession number MT509851.1. Further, the effect of GS-BC on ARN7 growth was assessed via infusion of 2.5 and 5% GS-BC in tryptone soy broth. When compared with the control, GS-BC infusion had a significant positive effect on ARN7 growth. However, 5% GS-BC treatment showed a maximum increase in the growth of ARN7 (Figure S2) demonstrating that GC-BS creates conditions favorable for ARN7 growth through its labile C, alkaline pH, porous structures, etc. [14,15].

### 2.3. Effect of ARN7 and GS-BC on Z. mays under HM and Drought Stress Condition

#### 2.3.1. Effect on Growth Parameters

The effect of ARN7 and GS-BC (sole and combined) on the growth of *Z. mays* was evaluated under different stress conditions including no stress, HM stress, drought stress, and combined (HM+drought) stress (Table 1).

Comparing the results with the different treatments, it becomes evident that the ARN7 treatment consistently led to improvement in shoot length, root length, fresh weight, and dry weight as compared to the control plants. Similarly, GS-BC treatment also exhibited positive effects on the growth parameters, although these effects were slightly less pronounced compared to the ARN7 treatment. Notably, the combined application of GS-BC and ARN7 resulted in further enhancement in growth parameters compared to the individual treatments, indicating a synergistic effect. Among the stress treatments, the combined stress (HM+drought) had notably more adverse effects on control plants which was consistent with findings of earlier works [17,20]. However, treatment with ARN7 and GS-BC had a significant positive effect on *Z. mays*, leading to an increase in shoot length, root length, fresh weight, and dry weight even under combined HM+drought stress conditions. For example, under the combined stress condition, ARN7 inoculation led to a 31% increase in shoot length and a remarkable increase (61%) in root length. Similarly, the combined GS-BC and ARN7 treatment showed maximum beneficial effects on plant growth under HM+drought stress, which increased shoot length, root length, fresh weight, and dry weight by 48, 98, 53, and 45%, respectively, as compared with control. The improvement in plant growth parameters as the consequence of ARN7 treatment align with prior findings [18,20,27], indicating that the PGP metabolites of ARN7, including siderophores, IAA, exopolysaccharides, and phosphate solubilization, contribute to the enhancement of *Z. mays* growth under HM and drought stress. On the other hand, biochar has also been extensively investigated as a soil amendment to boost plant growth and enhance soil properties [10,13]. It has been shown to enhance nutrient availability, water retention capacity, and soil structure, all of which positively influence plant growth and stress resilience [28]. The maximum plant beneficial effect observed in ARN7 + GS-BC treatment can be attributed to the synergistic effects of biochar and PGPR. In general, biochar provides a stable environment for microbial growth and survival, thereby improving their colonization and subsequent PGP activity [29,30].

#### 2.3.2. Effect on Physiological Parameters

**Figure 2:**
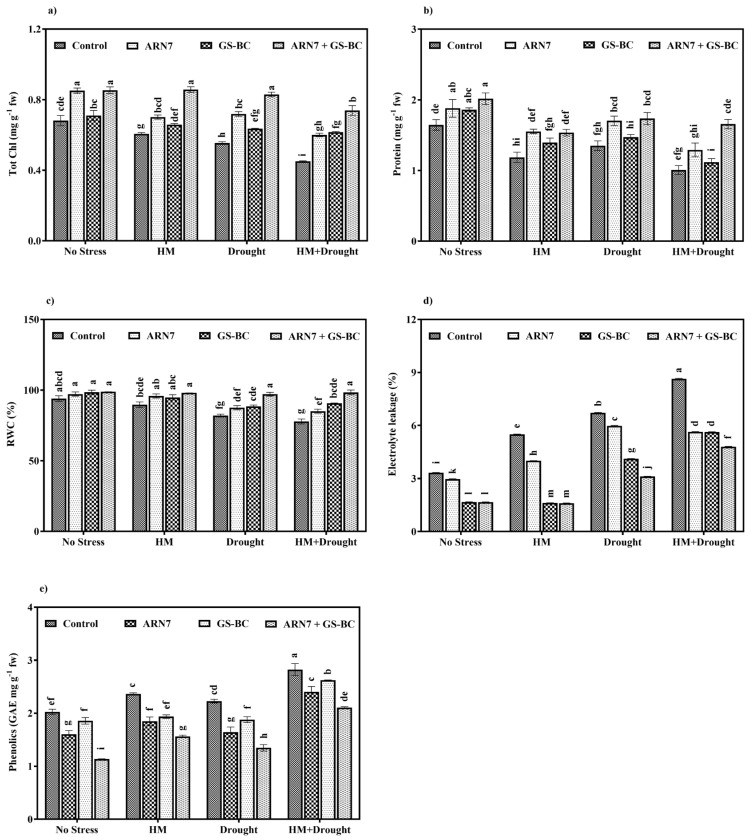
Effect of ARN7 and groundnut shell biochar on (**a**) total chlorophyll, (**b**) total soluble proteins, (**c**) relative water content (RWC), (**d**) electrolyte leakage (EL), and (**e**) phenolics of *Z. mays* grown under HM, drought, or HM+drought stress condition. Bars indexed with different alphabets are significantly different among the treatments tested according to HSD Tukey test at *p* < 0.05. fw = fresh weight.

Physiological parameters, including total chlorophyll, total soluble proteins, relative water content (RWC), electrolyte leakage (EL), and phenolics, were assessed in *Z. mays* leaves to evaluate the impact of ARN7 and GS-BC treatments under HM and drought stress conditions. The chlorophyll content, soluble protein levels, and RWC exhibited significant changes, displaying a notable decrease in untreated plants subjected to HM, drought, or HM+drought stress conditions. However, application of ARN7 and GS-BC had a significant positive effect on *Z. mays*, leading to an increase in the aforementioned content, even under HM, drought, and combined HM+drought stress conditions (Figure 2a–c). In general, the abiotic stresses, including HM and drought, can induce a decline in chlorophyll, protein, and RWC within plants by affecting critical factors such as chloroplast structure, chlorophyll synthesis, protein synthesis, turgor pressure, root growth, nutrient uptake, water availability, water uptake, and transport mechanisms [17,31,32]. On the other hand, several recent studies demonstrated that the application of PGPR and/or biochar in HM-contaminated or drought soils enhanced plant photosynthetic efficiency, protein synthesis, and RWC by altering osmotic potential, the accumulation of osmolytes, and nutrient absorption and attributed these beneficial effects to the inherent PGP traits of PGPR (IAA, siderophore production, phosphate solubilization, etc.,) and the properties of biochar (water holding capacity, surface area, etc.) [20,31,33]. Recently, Vishnupradeep et al. [17] and Saikia et al. [34] have found that inoculation with PGPR increased chlorophyll and cellular water contents by enhancing gaseous exchange, fluorescence parameters, protein structural stability, osmolyte production, and nutrient absorption under drought and HM stress. Similarly, Naveed et al. [33] and Danish and Zafar-ul-Hye [35] have documented the beneficial effect of biochar on plant chlorophyll, protein, and RWC through an improved water holding ability of the soil and nutrient absorption of plants under HM and drought stress conditions. Upon comparing the effects of ARN7 and GS-BC treatments in this study, ARN7 treatment had a more pronounced effect in enhancing leaf chlorophyll, soluble protein, and RWC content. However, the maximum increase in leaf chlorophyll, soluble protein, and RWC content was found as the result of combined application (ARN7 + GS-BC) under all stress conditions. For example, the combined application (ARN7 + GS-BC) resulted in an increase in leaf chlorophyll content by 64%, soluble protein by 65%, and RWC by 26% in comparison to control plants under HM+drought stress. A similar observation was documented by Naveed et al. [33], where the combined application of *Burkholderia phytofirmans* PsJN and biochar improved the aforementioned parameters to a greater extent than the sole application, suggesting the cumulative impact of biochar and PGPR on improving soil properties, stress tolerance, and nutrient supply to the plants. In contrast, a divergent trend was observed in the EL and phenolic content of *Z. mays* leaves. While the control plants showed an increase in EL and phenolic content under HM (67 and 14%, respectively), drought (103 and 10%, respectively) and combined (160 and 40%, respectively) stress conditions, the application of ARN7 and GS-BC led to a reduction in EL and phenolic content regardless of the stress conditions (Figure 2d,e) suggesting that ARN7 and GS-BC could lessen the HM and drought stress effect and improve membrane integrity.

#### 2.3.3. Effect on Stress-Related Metabolites and Antioxidant Activity

**Figure 3:**
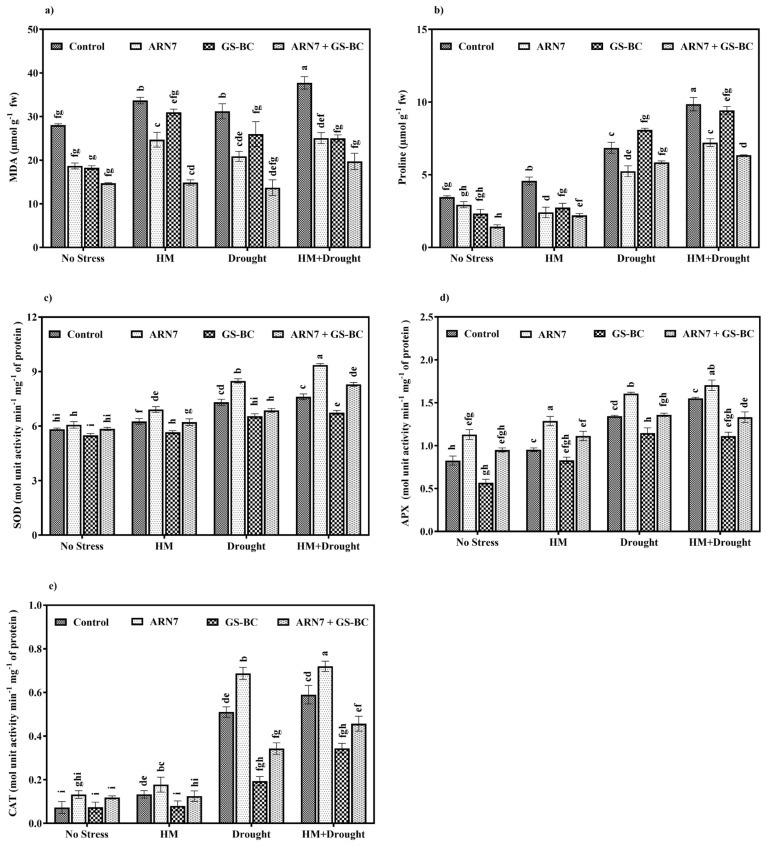
Effect of ARN7 and GS-BC on (**a**) malondialdehyde (MDA) content, (**b**) proline, (**c**) superoxide dismutase (SOD), (**d**) ascorbate peroxidase (APX), and (**e**) catalase (CAT) activity in *Z. mays* grown under HM, drought, or HM+drought stress conditions. Bars indexed with different alphabets are significantly different among the treatments tested according to HSD Tukey test at *p* < 0.05. fw = fresh weight.

To elucidate the role of ARN7 and GS-BC in enhancing plant resilience against HM and drought stress, we conducted a comprehensive analysis focusing on stress-related metabolites and antioxidant activities, including MDA, proline, SOD, APX, and CAT. The outcomes reveal that HM and drought stress triggered severe oxidative stress in control plants as HM, drought, and combined stress conditions increased the stress biomarker MDA by 141, 133, and 175%, respectively (Figure 3a). Conversely, treatment with ARN7 and GS-BC led to a substantial reduction in MDA content. Specifically, under the combined stress condition, ARN7, GS-BC, and ARN7 + GS-BC treatments decreased MDA content by 58, 67, and 67%, respectively. Remarkably, the sole application of GS-BC exhibited the most pronounced decrease compared to the ARN7 treatment (Figure 3a). This suggests that GS-BC application mitigates the harmful effects (oxidative damage) of HM and drought stress through the reduction in HM phytoavailability and the enhancement of soil biological properties, water holding capacity, etc. [36,37].

Plants adopt a multitude of defense strategies to cope with various stresses in their environment. The accumulation of proline is believed to play a very intriguing role in HM chelation, antioxidative defense, intracellular redox homeostasis, water potential, osmotic adjustment, etc., thereby conferring HM and drought stress tolerance [19]. Our findings also demonstrate that, irrespective of ARN7 and GS-BC treatment, the concentration of proline in *Z. mays* leaves increased under all stress conditions compared to nonstress control plants. However, the accumulation of proline under HM, drought, and HM+drought stress conditions was significantly lower in ARN7- and GS-BC-treated plants compared to their respective control counterparts (Figure 3b). Similar observations were previously documented by Anbuganesan et al. [7] and Tripti et al. [16] where they noted reduced proline content upon the application of biochar and PGP bacteria under HM and drought stress conditions and suggested that treatment with biochar and PGP bacteria cumulatively reduced the stress effects caused by HM and drought.

Furthermore, the results of antioxidant activity analysis revealed that HM, drought, and combined stress conditions increased SOD, APX, and CAT activities in *Z. mays* irrespective of biochar and ARN7 treatment. Interestingly, the sole application of ARN7 further increased the SOD, APX, and CAT activity in all stress condition. For instance, under the HM+drought condition, ARN7 inoculation increased the activities of SOD, APX, and CAT by 23, 28, and 61%, respectively, as compared to corresponding control plants. In contrast, the application of GS-BC alone or in combination decreased the activities of these antioxidants under all stress conditions (Figure 3c–e). Our findings suggest that ARN7 inoculation induces an antioxidant-mediated tolerance mechanism in plants to counteract stress effects. PGPR have the capability to upregulate antioxidant genes (SOD, APX, and CAT) during stress conditions, thereby enhancing antioxidant activity and reducing ROS-mediated oxidative damage [20]. On the other hand, biochar application may alleviate toxic effects by modifying soil physicochemical properties and HM availability which provide a conducive habitat for plants [38], leading to reduced oxidative damage, decreased proline accumulation, and lowered antioxidant activity [19]. The findings from the MDA analysis (Figure 3a) corroborate this explanation, displaying a decrease in MDA content upon GS-BC and ARN7+GS-BC treatments, indicating minimized stress exposure in plants.

#### 2.3.4. Effect on HM Accumulation

**Figure 4:**
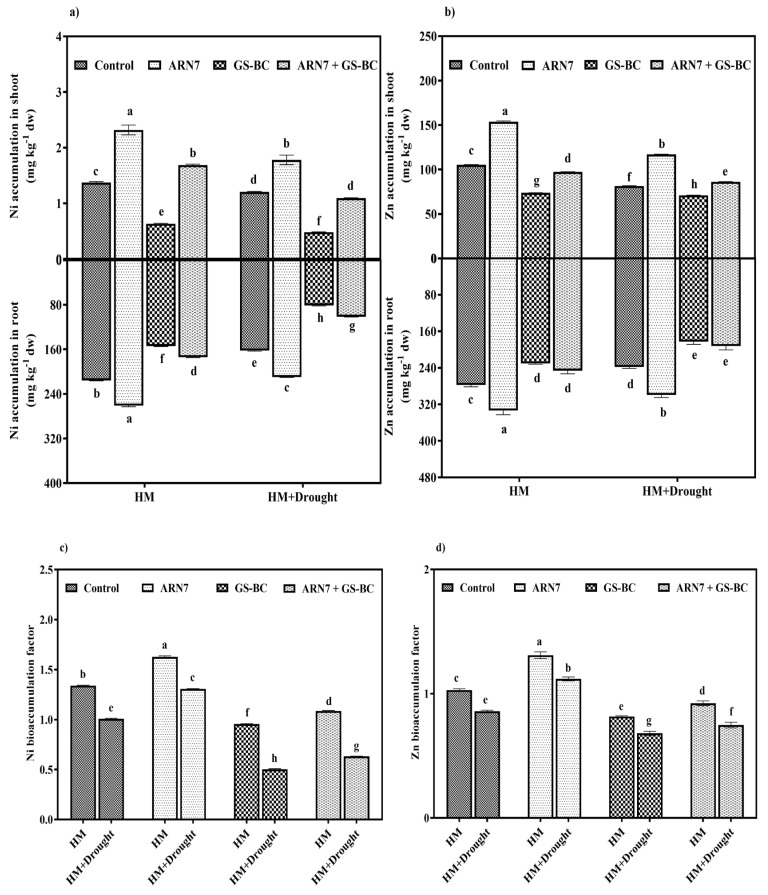
Effect of ARN7 and GS-BC on (**a**) Ni accumulation, (**b**) Zn accumulation, (**c**) Ni bioaccumulation factor, and (**d**) Zn bioaccumulation factor in *Z. mays* grown under HM or HM+drought stress conditions. Bars indexed with different alphabets are significantly different among the treatments tested according to HSD Tukey test at *p* < 0.05. dw = dry weight.

The results indicate that ARN7 treatment led to an increase in Ni and Zn accumulation in both the shoot and root tissues of *Z. mays* grown in HM-contaminated soils irrespective of drought stress. However, the application of GS-BC, either alone or in combination with ARN7, suppressed the uptake of Ni and Zn. Notably, the highest reduction was observed with the sole application of GS-BC. For instance, under the HM+drought condition, GS-BC application reduced Ni and Zn contents in shoots by 58 and 13% and roots by 50 and 23%, respectively (Figure 4a,b). Moreover, the combined application of ARN7 and GS-BC demonstrated a synergistic effect, leading to lower HM accumulation as compared to sole ARN7 inoculation. A similar trend was observed for the BAF of Ni and Zn, where combined application of ARN7 and GS-BC reduced the BAF irrespective of drought stress condition (Figure 4c,d). This might be as a result of the involvement of functional groups in the biochar in HM immobilization, chelation, or complex formation, resulting in decreased HM bioavailability and its uptake by plants [39,40]. Our results are in agreement with the findings of Naveed et al. [33], who demonstrated that biochar amendment reduced Pb content in roots and shoots of *Vigna radiata*. Conversely, the increased HM uptake upon sole ARN7 treatment implies that PGPR play a role in enhancing HM solubilization and mobilization, subsequently elevating HM concentrations in plant tissues [18,41]. However, the potential toxic effects resulting from increased HM uptake due to ARN7 inoculation was counteracted by the excessive induction of plant antioxidants as evidenced by the significant increase in MDA level, SOD, APX, and CAT activities (Figure 3).

### 2.4. Effect of ARN7 and GS-BC on Rhizospheric Soil under HM and Drought Condition

#### Effect on Soil Enzymes

**Figure 5:**
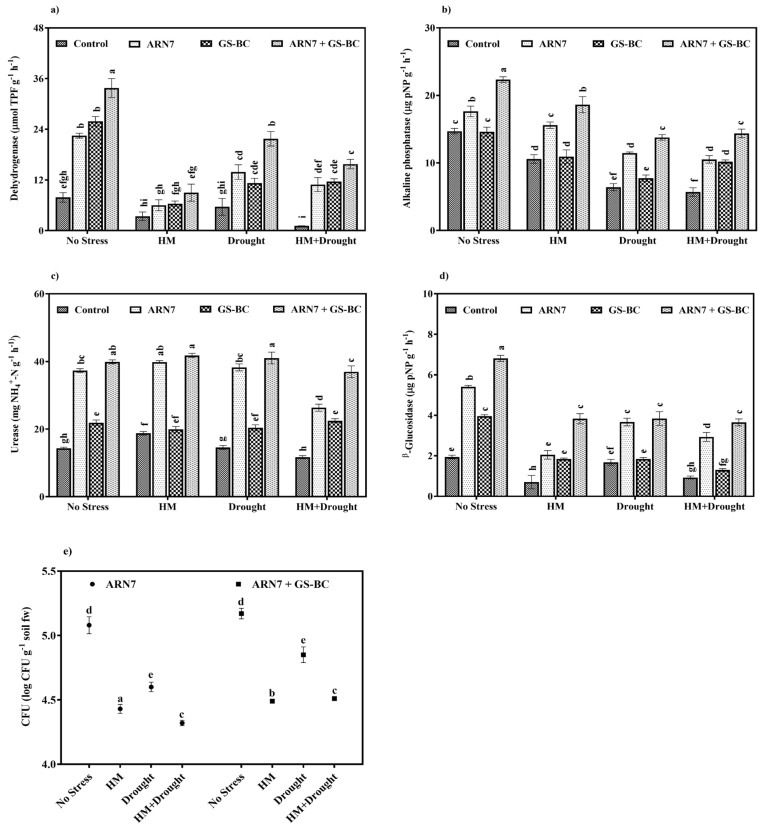
Effect of ARN7 and GS-BC on (**a**) dehydrogenase, (**b**) alkaline phosphatase, (**c**) urease, (**d**) β-glucosidase, and (**e**) colonization density of ARN7 in rhizosphere soil under HM, drought, or HM+drough stress conditions. Bars indexed with different alphabets are significantly different among the treatments tested according to HSD Tukey test at *p* < 0.05.

Soil enzymatic activity is considered to be one of the potential indicators of soil quality, which plays an inevitable role in maintaining soil fertility and nutrient cycling. To elucidate the impact of PGPR and biochar on soil enzymes, we analyzed the activity of soil dehydrogenase, alkaline phosphatase, urease, and β-glucosidase under conditions of HM, drought, and simultaneous stress. According to previous reports [42,43], HM and drought could inhibit soil enzyme activity either directly or indirectly by changing soil pH, moisture, and microbial colonization and their activity. Our study also confirmed this statement where we found that the activity of soil dehydrogenase, alkaline phosphatase, urease, and β-glucosidase were reduced under HM, drought, and combined stress conditions (Figure 5a–d). In particular, under the HM+drought stress condition, the aforementioned enzyme activities were decreased by 87, 61, 18, and 52%, respectively, in the rhizosphere soil of control plants. Therefore, our study postulated that the presence of HM and drought inhibit soil enzyme activity, probably due to the severe stress affecting the rhizosphere’s microbial population density, survival, and colonization potential.

The application of ARN7 and GS-BC improved the activity of dehydrogenase, alkaline phosphatase, urease, and β-glucosidase irrespective of stress condition. Compared to sole application, the combined application had a significant effect on these enzyme activities. For instance, under pristine condition, the activity of soil dehydrogenase, alkaline phosphatase, urease, and β-glucosidase were generally higher in ARN7 + GS-BC treatment, where it improved the activity by 328, 52, 178, and 251%, respectively, as compared to the control treatment (Figure 5a–d). Further, in the combined stress condition, the ARN7 + GS-BC treatment improved these enzyme activities by several fold, suggesting that the combined application significantly improved the microbial activity in the rhizosphere soil by improving soil physical, chemical, and biological properties. Similar results have been reported by Haroun et al. [10], Ma et al. [37], and Jabborova et al. [44], where the application of PGPR and biochar improved the activity of soil enzymes under HM and drought stress conditions. Several mechanisms have been explained [14,45,46] which correlate the application of BC and/or PGPR with their effect on soil enzymes under adverse environmental condition. For instance, Jin et al. [45] found that the application of biochar improved root colonization of the beneficial microbes in Cd-contaminated soil and suggested that the alteration of plant root exudation patterns due to biochar amendment could facilitate microbial colonization by acting as a signaling molecule and a nutrient source. Similarly, Ning et al. [46] also reported a positive correlation between root colonization and biochar + *Pseudomonas* sp. treatment. In general, the application of BC improves soil nutrient retention potential (P, K, and Carbon), mineralization, and respiration, which favors the colonized microbes increased density and survivability, as well as the activities of indigenous soil microbes [14,15]. Furthermore, biochar also decreases the adverse effect of HM and drought on the microbial population by creating a favorable microhabitat through reducing HM bioavailability and toxicity and improving soil moisture dynamics [45,47]. Therefore, in the present study, the increased soil enzyme activity was probably attributed to the increased density and colonization of ARN7 due to the addition of GS-BC. To support this hypothesis, we analyzed the colonization density of ARN7 in rhizosphere soil with/without GS-BC application under HM and drought stress conditions. The result revealed that the strain ARN7 could efficiently colonize the rhizosphere soil of *Z. mays* irrespective of HM and/or drought stress conditions (Figure 5e). However, as compared with the nonstressed condition, though a slight decrease in colonization was found in HM, drought, and combined stress conditions, GS-BC application under the aforementioned stress conditions improved the colonization efficacy of ARN7 indicating that GS-BC could protect the ARN7 from HM and drought stress by changing soil properties, providing nutrients, retaining soil moisture, and reducing HM toxicity and availability [7,12,15]. Therefore, the increased plant growth and physiological attributes caused by ARN7 + GS-BC treatment under HM, drought, or HM+drought stress highlights that GS-BS could improve soil quality including enzymatic activity and ARN7 colonization, growth, and PGP activity, thereby mitigating the negative effects of HM and drought stress in plants.

## 3. Materials and Methods

### 3.1. Preparation and Characterization of Groundnut Shell Biochar

The biochar was produced from readily available groundnut shell waste. The groundnut shell was collected, cleaned, oven dried (60 °C), crushed, and converted into biochar through pyrolysis at 400 °C for 90 min using a muffle furnace (I-Therm A1-7981) under limited oxygen conditions [48]. The produced groundnut shell biochar (GS-BC) was cooled at room temperature, sieved (2 mm), and characterized for physicochemical parameters. The pH and EC were analyzed in a 1:10 (*w*/*v*) ratio of GS-BC to deionized water. Additionally, CEC [49], moisture content [50], organic content [51], volatile matter, ash content [50], and yield percentage [52] were determined. The surface area of GS-BC was determined through the Brunauer–Emmett–Teller (BET) analysis (QuantachromeTouchWin v1.22, Boynton Beach, FL, USA). The elemental composition of GS-BC was determined using a CHNS elemental analyzer (SKALAR, Breda, The Netherlands). The surface morphology of GS-BC was examined with a scanning electron microscope (SEM) (Zeiss EVO MA 18 with Oxford EDS, Jena, Germany), and functional groups were examined with a Fourier transform infrared (FTIR) spectrometer (Shimadzu Prestige 20 FT-IR Spectrometer, Europe). The concentrations of K and Na were determined using a flame photometer (Accumax AIO-671, New Delhi, India). The other elements, including Mg, Al, Cu, Fe, Mn, Zn, Cd, Cr, Ni, and As, were analyzed with inductively coupled plasma mass spectrometry (ICP-MS) (Thermo Fisher Scientific^®^, Waltham, MA, USA).

### 3.2. Isolation, Characterization, and Identification of PGPR

The bacterial strain ARN7 was isolated from the rhizosphere soil of *Azadirachta indica*, Magnesite Mines, Salem, India. The concentrations of HM in the rhizospheric soil, including Ni, Cu, Zn, Cr, and Cd, were 175, 41, 64, 115, and 5.5 mg kg^−1^, respectively. To assess the maximum HM tolerance of ARN7, the strain was inoculated on LB agar media supplemented with increasing concentrations of Ni, Cu, Zn, Cr, and Cd (0–500 mg L^−1^) [53]. Furthermore, the drought tolerance of ARN7 was evaluated by subjecting it to tryptone yeast-extract and glucose (TYEG) agar medium containing 15% (low) and 30% (moderately high) polyethylene glycol (PEG 6000), following the method described by Vishnupradeep et al. [17]. An in vitro phytagar assay was carried out to assess the plant growth-promoting potential of ARN7 on *Z. mays* as per the protocol described by Ma et al. [54]. For in vitro studies, healthy seeds procured from the local market were surface sterilized, inoculated with ARN7, and allowed to grow for 20 d as described previously [7,20].

To characterize the PGP attributes of ARN7 under various stress conditions, including HM (Ni-150 mg L^−1^ [NiSO_4_.6H_2_O] + Zi—300 mg L^−1^ [ZnSO_4_.7H_2_O]), drought (15 and 30% of PEG 6000), and their combinations, quantitative analyses, including the production of IAA [55], siderophore [56], hydroxymate type siderophores [57], catechol type siderophores [58], phosphate solubilization [59], and exopolysaccharides (EPS) production [60], were performed. In addition, the effect of GS-BC on the growth rate of ARN7 was investigated by inoculating the strain in tryptone soya broth amended with 0, 2.5, and 5% GS-BC, and the colony-forming units (CFU) were determined at various time intervals. The strain was further identified as *Bacillus pseudomycoides* through 16S rRNA gene sequencing using the primers 27F (5′ AGAGTTTGATCTGGCTCAG 3′) and 1492R (5′ TACGGTACCTTGTTACGACTT 3′) [18] and the identification was confirmed through the BLASTn algorithm.

### 3.3. Pot Experiment

A controlled pot culture experiment was carried out in the laboratory at the Department of Environmental Sciences, Bharathiar University, Coimbatore, India. Garden soil (0–10 cm depth) from the department was utilized to perform the pot experiments and its physicochemical characteristic features were analyzed and reported [7]. The soil was sterilized to eliminate the existing bacterium and other microbes as detailed by Bruno et al. [20]. The experiments were carried out under the following conditions: (i) Nonstress, (ii) HM, (iii) drought, (iv) and HM + drought, as well as each with four treatments (control, ARN7, GS-BC, and ARN7+GS-BC). To prepare HM polluted soils, Ni (150 mg kg^−1^) and Zn (300 mg kg^−1^) were artificially spiked in the sterilized garden soil by mixing the aqueous solution of NiSO_4_.6H_2_O and ZnSO_4_.7H_2_O and left (4 weeks) aseptically for drying and HM stabilization. In the case of GS-BC treatment, 5% of GS-BC was applied to the soil, mixed properly and placed in the pot before sowing the seeds. As for the ARN7 treatment, surface sterilized seeds were soaked in a suspension of ARN7 (10^8^ CFU in sterile distilled water) for 2 h, while control seeds were soaked in sterile water. Prior to priming of the strain with seeds, ARN7 was marked with ampicillin (110 mg L^−1^) to analyze the density and colonization in the rhizosphere as described by Ma et al. [54]. Then, six seeds (ARN7 treated or untreated) were sown in pots containing 500 g of unpolluted, HM polluted, and/or GS-BC applied soil and were allowed to germinate under a natural day–night regime of 16:8 h at 36/24 ± 1 °C (day/night). Initially, for the first 15 d, the soil moisture content was maintained using a soil moisture meter at 100% of field capacity as detailed by Vishnupradeep et al. [17]. After 15 d from emergence, the plants were thinned to three per pots and water limitation was imposed for the respective drought stress by maintaining the soil moisture content at 50% of field capacity for another 45 d. Each treatment was conducted in triplicates.

### 3.4. Analyses of Z. mays Growth Parameters

After completion of the experiment, the plants were uprooted, washed several times with distilled water to eliminate the adhered soil debris, and air dried. Then, the plant growth parameters, including shoot length, root length, fresh weight, and dry weight, were analyzed.

### 3.5. Analyses of Z. mays Physiological Parameters

The chlorophyll content in *Z. mays* leaves was assessed by extracting pigments in 5 mL of acetone (80%) following the methodology outlined by Ni et al. [61]. Protein content was estimated using the Bradford method [62]. The relative water content (RWC) was determined as per the procedure described by Barrs and Weatherley [63]. Electrolyte leakage (EL) from leaves was quantified as detailed by Campos et al. [64]. The total phenol content (TPC) was analyzed following the methodology outlined by Singleton et al. [65].

### 3.6. Effect on Stress-Related Metabolites and Antioxidant Activity

The changes in lipid peroxidation level were determined by analyzing the malondialdehyde (MDA) content of *Z. mays* leaves under different stress conditions according to the standard method described by Heath and Packer [66]. Free proline content was quantified through a ninhydrin reaction as per the procedure described by Bates et al. [67]. For antioxidant enzyme activity analysis, fresh leaf samples of about 0.5 g were homogenized with 10 mL of 0.1 M ice-cold phosphate buffer (pH 7.0) and centrifuged (10,000 rpm, 1 min, 4 °C), and the supernatant collected was used for the assay of SOD [68], APX [69], and CAT activity [70].

### 3.7. Analysis of Zn and Ni Accumulation

For determination of Zn and Ni content in the shoot and root tissues of *Z. mays*, the samples were rinsed thoroughly with distilled water, oven dried, ground, and digested using aqua regia (HNO_3_: HCl in 4:1 ratio) [54]. The concentrations of Zn and Ni were measured with ICP-MS. Further, the BAF of Zn and Ni was calculated by dividing the concentration of a specific HM in plant tissues with the concentration of the respective HM in the soil [71].

### 3.8. Soil Enzymatic Activity and ARN7 Colonization

The rhizospheric soil of *Z. mays* was collected after the pot experiment to analyze the activities of soil enzymes and to examine colonization and density of the introduced strain ARN7. The dehydrogenase activity of the soil was assayed by analyzing the quantity of triphenylformazan (TPF) formation according to the standard method [72]. Alkaline phosphatase activity was evaluated via estimation of p-nitrophenol (pNP) release from p-nitrophenyl phosphate [73]. Urease activity was determined spectrophotometrically at 578 nm by measuring the NH_4_-N colored complex using a urea solution (10%) as the substrate [74]. β-glucosidase activity was evaluated using ρ-nitrophenyl- β-D-glucoside as a substrate [75]. Further, the colonization potential of the introduced ARN7 was evaluated as described previously [7]. Briefly, 1 g of soil was mixed with 50 mL of sterile, distilled water and plated on an LB medium amended with 110 mg L^−1^ ampicillin. After 5 d of incubation at 35 °C, the strains were reisolated, and HM-resistant strains were identified for colony characteristics and HM-resistance (Ni and Zn) against the parent strains.

### 3.9. Statistical Analyzes

The values are reported as mean ± standard deviation based on three replicates. The results were analyzed by using one way analysis of variance. Comparisons between treatment means were assessed with Tukey’s HSD test at *p* < 0.05.

## 4. Conclusions

Multiple abiotic stressors including HM and drought exhibit a devastating impact on plant growth and crop productivity as compared with individual stress. In the present study, HM, drought, or HM+drought stress reduced *Z. mays* growth, physiological parameters (total chlorophyll, proteins, phenolics, and RWC), antioxidant levels (SOD, APX, and CAT), and increased EL, phenolics, MDA, proline, and HM accumulation and bioaccumulation factor. However, treatment with PGPR *Bacillus pseudomycoides* strain ARN7 and groundnut shell biochar improved plant growth under HM and drought stress. Particularly, the combined application of ARN7 and GS-BC was found to be most effective for improving plant growth, physiological parameters (total chlorophyll, proteins, phenolics, and RWC), antioxidant levels (SOD, APX, and CAT), and reducing EL, phenolics, MDA, proline, and HM accumulation and bioaccumulation factor even under HM+drought stress. Additionally, the potential of GS-BC to improve ARN7 colonization and soil enzymatic activities, including dehydrogenase, urease, alkaline phosphatase, and β-glucosidase, may also have contributed to the improvement of soil quality, plant growth, and tolerance to HM and drought stress. Our findings demonstrated that the combined utilization of PGPR and biochar have substantial potential to improve plant growth, HM and drought stress tolerance, and phytostabilization potential. However, further studies including the analysis of the long-term effects of PGPR and biochar on plant growth and physiological response, molecular interactions between PGPR, biochar, and plants, etc., are required in order to utilize such a combined treatment in water-deficient HM-contaminated field soils.

## Figures and Tables

**Table 1:** Effect of ARN7 and GS-BC on the growth attributes of *Z. mays* under HM and drought stress.

	Treatment Abbreviation	Shoot Length (cm)	Root Length (cm)	Fresh Weight (g/plant)	Dry Weight (g/plant)
**No Stress**	Control	18.2 ± 0.96 ^cdef^	26.0 ± 1.46 ^fg^	3.25 ± 0.19 ^bcd^	0.48 ± 0.08 ^de^
	ARN7	23.5 ± 2.13 ^ab^	43.1 ± 1.25 ^b^	4.16 ± 0.41 ^a^	0.82 ± 0.05 ^ab^
	GS-BC	19.5 ± 0.31 ^abcd^	39.0 ± 2.16 ^bc^	3.44 ± 0.17 ^abc^	0.79 ± 0.02 ^ab^
	ARN7 + GS-BC	21.9 ± 2.05 ^abc^	55.9 ± 2.05 ^a^	4.20 ± 0.22 ^a^	0.89 ± 0.08 ^a^
**HM**	Control	14.6 ± 1.08 ^ef^	20.9 ± 0.66 ^gh^	2.25 ± 0.40 ^f^	0.47 ± 0.05 ^de^
	ARN7	21.2 ± 2.71 ^abc^	38.8 ± 2.59 ^bc^	3.53 ± 0.38 ^abc^	0.79 ± 0.06 ^ab^
	GS-BC	20.7 ± 0.87 ^abcd^	34.1 ± 2.01 ^cd^	2.95 ± 0.07 ^cdef^	0.71 ± 0.05 ^bc^
	ARN7 + GS-BC	24.0 ± 2.80 ^a^	40.4 ± 2.35 ^b^	3.95 ± 0.18 ^ab^	0.79 ± 0.05 ^ab^
**Drought**	Control	16.2 ± 1.03 ^def^	20.5 ± 0.72 ^gh^	2.51 ± 0.07 ^def^	0.41 ± 0.04 ^de^
	ARN7	20.5 ± 1.86 ^abcd^	32.5 ± 2.08 ^de^	3.80 ± 0.38 ^ab^	0.77 ± 0.09 ^ab^
	GS-BC	19.0 ± 1.15 ^bcde^	27.4 ± 3.59 ^ef^	3.2 ± 0.05 ^bcde^	0.66 ± 0.02 ^bc^
	ARN7 + GS-BC	21.2 ± 1.20 ^abc^	39.5 ± 1.31 ^bc^	3.46 ± 0.23 ^abc^	0.72 ± 0.05 ^abc^
**HM+** **Drought**	Control	13.8 ± 1.11 ^f^	19.5 ± 0.55 ^h^	2.45 ± 0.04 ^ef^	0.40 ± 0.06 ^e^
	ARN7	18.1 ± 0.82 ^cdef^	31.3 ± 1.53 ^def^	3.54 ± 0.21 ^abc^	0.49 ± 0.08 ^de^
	GS-BC	17.4 ± 0.67 ^cdef^	27.3 ± 1.80 ^ef^	2.63 ± 0.38 ^def^	0.35 ± 0.02 ^e^
	ARN7 + GS-BC	20.4 ± 0.79 ^abcd^	38.7 ± 0.91 ^bc^	3.74 ± 0.14 ^ab^	0.58 ± 0.06 ^cd^

Bars (±) represent standard deviations of three replicates. Bars indexed with the same letter are not significantly different between treatments according to the HSD Tukey test at *p* < 0.05.

## Data Availability

Data are contained within the article or Supplementary Materials.

## Supplementary Materials

The following supporting information can be downloaded at: https://www.mdpi.com/article/10.3390/plants13081143/s1. Table S1. Physicochemical characteristics of groundnut shell biochar; Table S2. Heavy metal stress tolerance and PGP features of ARN7; Figure S1. Phylogenetic tree showing the relationship of partial 16S rRNA gene sequences of ARN7 with other related sequences obtained from the NCBI database. The tree was clustered with the neighbor-joining method using the MEGA 11 package; Figure S2. Growth pattern of ARN7 in tryptone soy broth medium supplemented with 0, 2.5, or 5% biochar.

## References

[1] Feng D., Wang R., Sun X., Liu L., Liu P., Tang J., Zhang C., Liu H. (2023). Heavy Metal Stress in Plants: Ways to Alleviate with Exogenous Substances. Sci. Total Environ..

[2] Stolpe C., Kramer U., Mullera C. (2017). Heavy Metal (Hyper) Accumulation in Leaves of *Arabidopsis halleri* is Accompanied by a Reduced Performance of Herbivores and Shifts in Leaf Glucosinolate and Element Concentrations. Environ. Exp. Bot..

[3] Rashid U., Yasmin H., Hassan M.N., Naz R., Nosheen A., Sajjad M., Ilyas N., Keyani R., Jabeen Z., Mumtaz S. (2022). Drought-tolerant Bacillus megaterium Isolated from Semi-Arid Conditions Induces Systemic Tolerance of Wheat under Drought Conditions. Plant Cell Rep..

[4] Islam M., Sandhi A. (2023). Heavy Metal and Drought Stress in Plants: The Role of Microbes—A Review. Gesunde Pflanz..

[5] Harindintwali J.D., Zhou J., Yang W., Gu Q., Yu X. (2020). Biochar-Bacteria-Plant Partnerships: Eco-Solutions for Tackling Heavy Metal Pollution. Ecotoxicol. Environ. Saf..

[6] Lei S., Shi Y., Qiu Y., Che L., Xue C. (2019). Performance and Mechanisms of Emerging Animal-Derived Biochars for Immobilization of Heavy Metals. Sci. Total Environ..

[7] Anbuganesan V., Vishnupradeep R., Mehnaz N., Kumar A., Freitas H., Rajkumar M. (2024). Synergistic Effect of Biochar and Plant Growth Promoting Bacteria Improve the Growth and Phytostabilization Potential of *Sorghum bicolor* in Cd and Zn Contaminated Soils. Rhizosphere.

[8] Zhu Y., Wang H., Lv X., Zhang Y., Wang W. (2020). Effects of Biochar and Biofertilizer on Cadmium-Contaminated Cotton Growth and the Antioxidative Defense System. Sci. Rep..

[9] Kamran M., Malik Z., Parveen A., Zong Y., Abbasi G.H., Rafiq M.T., Shaabane M., Mustafa A., Bashir S., Rafay M. (2019). Biochar Alleviates Cd Phytotoxicity by Minimizing Bioavailability and Oxidative Stress in Pakchoi (*Brassica chinensis* L.) Cultivated in Cd-Polluted Soil. J. Environ. Manag..

[10] Haroun M., Xie S., Awadelkareem W., Wang J., Qian X. (2023). Influence of Biofertilizer on Heavy Metal Bioremediation and Enzyme Activities in the Soil to Revealing the Potential for Sustainable Soil Restoration. Sci. Rep..

[11] Guo X., Li H. (2019). Effects of Iron-Modified Biochar and AMF Inoculation on the Growth and Heavy Metal Uptake of *Senna occidentalis* in Heavy Metal-Contaminated Soil. Pol. J. Environ. Stud..

[12] Lalarukh I., Amjad S.F., Mansoora N., Al-Dhumri S.A., Alshahri A.H., Almutari M.M., Alhusayni F.S., Al-Shammari W.B., Poczai P., Abbas M.H.H. (2022). Integral Effects of Brassinosteroids and Timber Waste Biochar Enhances the Drought Tolerance Capacity of Wheat Plant. Sci. Rep..

[13] Zulfiqar B., Raza M.A.S., Saleem M.F., Aslam M.U., Iqbal R., Muhammad F., Amin J., Ibrahim M.A., Khan I.H. (2022). Biochar Enhances Wheat Crop Productivity by Mitigating the Effects of Drought: Insights into Physiological and Antioxidant Defense Mechanisms. PLoS ONE.

[14] Waqar A., Bano A., Ajmal M. (2022). Effects of PGPR Bioinoculants, Hydrogel and Biochar on Growth and Physiology of Soybean under Drought Stress. Commun. Soil Sci. Plant Anal..

[15] Zaheer M.S., Ali H.H., Soufan W., Iqbal R., Habib-ur-Rahman M., Iqbal J., Israr M., El Sabagh A. (2021). Potential Effects of Biochar Application for Improving Wheat (*Triticum aestivum* L.) Growth and Soil Biochemical Properties under Drought Stress Conditions. Land.

[16] Tripti, Kumar A., Maleva M., Borisova G., Rajkumar M. (2023). Amaranthus Biochar-Based Microbial Cell Composites for Alleviation of Drought and Cadmium Stress: A Novel Bioremediation Approach. Plants.

[17] Vishnupradeep R., Bruno L.B., Taj Z., Karthik C., Challabathula D., Tripti, Kumar A., Freitas H., Rajkumar M. (2022). Plant Growth Promoting Bacteria Improve Growth and Phytostabilization Potential of *Zea mays* under Chromium and Drought Stress by Altering Photosynthetic and Antioxidant Responses. Environ. Technol. Innov..

[18] Bruno L.B., Anbuganesan V., Karthik C., Tripti, Kumar A., Banu J.R., Rajkumar M. (2021). Enhanced Phytoextraction of Multi-Metal Contaminated Soils under Increased Atmospheric Temperature by Bioaugmentation with Plant Growth Promoting *Bacillus cereus*. J. Environ. Manag..

[19] Yildiztugay E., Ozfidan-Konakci C., Arikan B., Alp-Turgut F.N., Gulenturk C. (2023). The Regulatory Effects of Biochar on PSII Photochemistry, Antioxidant System and Nitrogen Assimilation in Lemna minor Exposed to Inorganic Pollutants, Arsenic and Fluoride. J. Environ. Chem. Eng..

[20] Bruno L.B., Karthik C., Ma Y., Kadirvelu K., Freitas H., Rajkumar M. (2020). Amelioration of Chromium and Heat Stresses in *Sorghum bicolor* by Cr^6+^ Reducing Thermotolerant Plant Growth Promoting Bacteria. Chemosphere.

[21] Gondek K., Mierzwa-Hersztek M., Kopec M., Sikora J., Głąb T., Szczurowska K. (2019). Influence of Biochar Application on Reduced Acidification of Sandy Soil, Increased Cation Exchange Capacity, and the Content of Available Forms of K, Mg, and P. Pol. J. Environ. Stud..

[22] Tao Q., Li B., Li Q., Han X., Jiang Y., Jupa R., Wang C., Li T. (2019). Simultaneous Remediation of Sediments Contaminated with Sulfamethoxazole and Cadmium using Magnesium-Modified Biochar Derived from *Thalia dealbata*. Sci. Total Environ..

[23] Yao Y., Gao B., Zhang M., Inyang M., Zimmerman A.R. (2012). Effect of Biochar Amendment on Sorption and Leaching of Nitrate, Ammonium, and Phosphate in a Sandy Soil. Chemosphere.

[24] Gai X., Wang H., Liu J., Zhai L., Liu S., Ren T., Liu H. (2014). Effects of feedstock and pyrolysis temperature on biochar adsorption of ammonium and nitrate. PLoS ONE.

[25] Yang Y., Piao Y., Wang R., Su Y., Liu N., Lei Y. (2022). Nonmetal Function Groups of Biochar for Pollutants Removal: A Review. J. Hazard. Mater. Adv..

[26] Vurukonda S.S.K.P., Vardharajula S., Shrivastava M., Skz A. (2016). Enhancement of Drought Stress Tolerance in Crops by Plant Growth Promoting Rhizobacteria. Microbiol. Res..

[27] Tripti, Kumar A., Kumar V., Anshumali, Bruno L.B., Rajkumar M. (2022). Synergism of Industrial and Agricultural Waste as a Suitable Carrier Material for Developing Potential Biofertilizer for Sustainable Agricultural Production of Eggplant. Horticulturae.

[28] Ren H., Li Z., Chen H., Zhou J., Lv C. (2022). Effects of Biochar and Plant Growth-Promoting Rhizobacteria on Plant Performance and Soil Environmental Stability. Sustainability.

[29] Malik L., Sanaullah M., Mahmood F., Hussain S., Siddique M.H., Anwar F., Shahzad T. (2022). Unlocking the Potential of Co-Applied Biochar and Plant Growth-Promoting Rhizobacteria (PGPR) for Sustainable Agriculture under Stress Conditions. Chem. Biol. Technol. Agric..

[30] Wang Y., Li W., Du B., Li H. (2021). Effect of Biochar Applied with Plant Growth-Promoting Rhizobacteria (PGPR) on Soil Microbial Community Composition and Nitrogen Utilization in Tomato. Pedosphere.

[31] Ashrafi M., Azimi-Moqadam M., Moradi P., Fard E.M., Shekari F., Kompany-Zareh M. (2018). Effect of Drought Stress on Metabolite Adjustments in Drought Tolerant and Sensitive Thyme. Plant Physiol. Biochem..

[32] Farooq M.A., Ali S., Hameed A., Bharwana S.A., Rizwan M., Ishaque W., Farid M., Mahmood K., Iqbal Z. (2016). Cadmium Stress in Cotton Seedlings: Physiological, Photosynthesis and Oxidative Damages Alleviated by Glycinebetaine. S. Afr. J. Bot..

[33] Naveed M., Mustafa A., Azhar S.Q., Kamran M., Zahir Z.A., Nunez-Delgado A. (2020). *Burkholderia phytofirmans* PsJN and Tree Twigs Derived Biochar Together Tetrieved Pb-Induced Growth, Physiological and Biochemical Disturbances by Minimizing its Uptake and Translocation in Mung Bean (*Vigna radiata* L.). J. Environ. Manag..

[34] Saikia J., Sarma R.K., Dhandia R., Yadav A., Bharali R., Gupta V.K., Saikia R. (2018). Alleviation of Drought Stress in Pulse Crops with ACC Deaminase Producing Rhizobacteria Isolated from Acidic Soil of Northeast India. Sci. Rep..

[35] Danish S., Zafar-ul-Hye M. (2019). Co-Application of ACC-Deaminase Producing PGPR and Timber-Waste Biochar Improves Pigments Formation, Growth and Yield of Wheat under Drought Stress. Sci. Rep..

[36] Ahmad M., Wang X., Hilger T.H., Luqman M., Nazli F., Hussain A., Zahir Z.A., Latif M., Saeed Q., Malik H.A. (2020). Evaluating Biochar-Microbe Synergies for Improved Growth, Yield of Maize, and Post-Harvest Soil Characteristics in a Semi-Arid Climate. Agronomy.

[37] Ma H., Wei M., Wang Z., Hou S., Li X., Xu H. (2020). Bioremediation of Cadmium Polluted Soil Using a Novel Cadmium Immobilizing Plant Growth Promotion Strain Bacillus sp. TZ5 Loaded on Biochar. J. Hazard. Mater..

[38] Alidou-Arzika I., Lebrun M., Miard F., Nandillon R., Baycu G., Bourgerie S., Morabito D. (2021). Assessment of Compost and Three Biochars Associated with *Ailanthus altissima* (Miller) Swingle for Lead and Arsenic Stabilization in a Post-Mining Technosol. Pedosphere.

[39] Karer J., Zehetner F., Dunst G., Fessl J., Wagner M., Puschenreiter M., Stapkevica M., Friesl-Hanl W., Soja G. (2018). Immobilisation of Metals in a Contaminated Soil with Biochar-Compost Mixtures and Inorganic Additives: 2-year Greenhouse and Field Experiments. Environ. Sci. Pollut. Res. Int..

[40] Tan Z., Wang Y., Zhang L., Huang Q. (2017). Study of the Mechanism of Remediation of Cd-Contaminated Soil by Novel Biochars. Environ. Sci. Pollut. Res. Int..

[41] Rojjanateeranaj P., Sangthong C., Prapagdee B. (2017). Enhanced Cadmium Phytoremediation of *Glycine max* L. Through Bioaugmentation of Cadmium-Resistant Bacteria Assisted by Biostimulation. Chemosphere.

[42] Qu Q., Wang Z., Gan Q., Liu R., Xu H. (2023). Impact of Drought on Soil Microbial Biomass and Extracellular Enzyme Activity. Front. Plant Sci..

[43] Tang B., Xu H., Song F., Ge H., Yue S. (2022). Effects of Heavy Metals on Microorganisms and Enzymes in Soils of Lead-Zinc Tailing Ponds. Environ. Res..

[44] Jabborova D., Kannepalli A., Davranov K., Narimanov A., Enakiev Y., Syed A., Elgorban A.M., Bahkali A.H., Wirth S., Sayyed R.Z. (2021). Co-Inoculation of Rhizobacteria Promotes Growth, Yield, and Nutrient Contents in Soybean and Improves Soil Enzymes and Nutrients under Drought Conditions. Sci. Rep..

[45] Jin X., Rahman M.K.U., Ma C., Zheng X., Wu F., Zhou X. (2023). Silicon Modification Improves Biochar’s Ability to Mitigate Cadmium Toxicity in Tomato by Enhancing Root Colonization of Plant-Beneficial Bacteria. Ecotoxicol. Environ. Saf..

[46] Ning Y., Xiao Z., Weinmann M., Li Z. (2019). Phosphate Uptake is Correlated with the Root Length of Celery Plants following the Association between Arbuscular Mycorrhizal Fungi, *Pseudomonas* sp. and Biochar with Different Phosphate Fertilization Levels. Agronomy.

[47] Liang C., Zhu X., Fu S., Mendez A., Gasco G., Paz-Ferreiro J. (2014). Biochar Alters the Resistance and Resilience to Drought in a Tropical Soil. Environ. Res. Lett..

[48] Sahoo S.S., Vijay V.K., Chandra R., Kumar H. (2021). Production and Characterization of Biochar Produced from Slow Pyrolysis of Pigeon Pea Stalk and Bamboo. Clean. Eng. Technol..

[49] Laird D., Fleming P., Ulery A.L., Drees R.L. (2008). Analysis of layer charge, cation and anion exchange capacities and synthesis of reduced charge clays. Methods of Soil Analysis: Mineralogical Methods.

[50] (1990). Standard Method for Laboratory Determination of Water (Moisture) Content of Soil and Rock.

[51] Nelson D.W., Sommers L.E., Sparks D.L., Page A.L., Helmke P.A., Loeppert R.H., Soltanpour P.N., Tabatabai M.A., Johnston C.T., Sumnner M.E. (1996). Total carbon, organic carbon and organic matter. Methods of Soil Analysis, Part 3. Chemical Methods.

[52] Sadaka S., Sharara M.A., Ashworth A., Keyser P., Allen F., Wright A. (2014). Characterization of Biochar from Switchgrass Carbonization. Energies.

[53] Sheng X., Xia J., Jiang C., He L., Qian M. (2008). Characterization of Heavy Metal-Resistant Endophytic Bacteria from Rape (*Brassica napus*) Roots and their Potential in Promoting the Growth and Lead Accumulation of Rape. Environ. Pollut..

[54] Ma Y., Rajkumar M., Luo Y.M., Freitas H. (2011). Inoculation of Endophytic Bacteria on Host and Non-Host Plants-Effects on Plant Growth and Ni Uptake. J. Hazard. Mater..

[55] Bric J.M., Bostock R.M., Silverstone S.E. (1991). Rapid In Situ Assay for Indole Acetic Acid Production by Bacteria Immobilized on a Nitrocellulose Membrane. Appl. Environ. Microbiol..

[56] Ghosh P.K., Sen S.K., Maiti T.K. (2015). Production and Metabolism of IAA by *Enterobacter* spp. (*Gamma proteobacteria*) Isolated from Root Nodules of a Legume *Abrus precatorius* L.. Biocatal. Agric. Biotechnol..

[57] Atkin C.L., Neilands J.B., Phaff H.J. (1970). Rhodotorulic Acid from Species of *Leucosporidium, Rhodosporidium, Rhodotorula, Sporidiobolus,* and *Sporobolomyces*, and a New Alanine-Containing Ferrichrome from Cryptococcus Melibiosum. J. Bacteriol..

[58] Arnow E. (1937). Colorimetric Determination of the Components of 3,4- Dihydroxyphenylalanine Tyrosine Mixtures. J. Biol. Chem..

[59] Pikovskaya R. (1948). Mobilization of Phosphorus in Soil in Connection with Vital Activity of Some Microbial Species. Mikrobiologiya.

[60] Rao B.P., Sudharsan K., Sekaran R.C.H., Mandal A.B. (2013). Characterization of Exopolysaccharide from Bacillus amyloliquefaciens BPRGS for its Bioflocculant Activity. Int. J. Eng. Res..

[61] Ni Z., Kim E., Chen E.Z. (2009). Chlorophyll and starch assays. Protoc. Exch. Nat..

[62] Bradford M.M. (1976). A Rapid and Sensitive Method for the Quantitation of Microgram Quantities of Protein Utilizing the Principle of Protein-Dye Binding. Anal. Biochem..

[63] Barrs H.D., Weatherley P.E. (1962). A Re-Examination of the Relative Turgidity Technique for Estimating Water Deficits in Leaves. Aust. J. Biol. Sci..

[64] Campos P.S., Quartin V., Ramalho J.C., Nunes M.A. (2003). Electrolyte Leakage and Lipid Degradation Account for Cold Sensitivity in Leaves of *Coffea* sp. Plants. J. Plant Physiol..

[65] Singleton V.L., Orthofer R., Lamuela-Raventos R.M. (1999). Analysis of Total Phenols and other Oxidation Substrates and Antioxidants by Means of Folin- Ciocalteu Reagent. Methods Enzymol..

[66] Heath R.L., Packer L. (1968). Photoperoxidation in Isolated Chloroplasts. I. Kinetics and Stoichiometry of Fatty Acid Peroxidation. Arch. Biochem. Biophys..

[67] Bates L.S., Waldren R.P., Teare I.D. (1973). Rapid Determination of Free Proline for Water Stress Studies. Plant Soil.

[68] Kono Y. (1978). Generation of Superoxide Radical during Auto Oxidation of Hydroxyl Amine and an Assay for Superoxide Dismutase. Arch. Biochem. Biophys..

[69] Chen G., Asada K. (1989). Ascorbate Peroxidase in Tea Leaves: Occurrence of Two Isoenzymes and the Differences in their Enzymatic and Molecular Properties. Plant Cell Physiol..

[70] Lata C., Jha S., Dixit V., Prasad M. (2011). Differential Antioxidative Responses to Dehydrationinduced Oxidative Stress in Core Set of Foxtail Millet Cultivars [*Setaria italica* (L.)]. Protoplasma.

[71] Wang S., Wu W., Liu F., Liao R., Hu Y. (2017). Accumulation of Heavy Metals in Soil Crop Systems: A Review for Wheat and Corn. Environ. Sci. Pollut. Res..

[72] Małachowska-Jutsz A., Matyja K. (2019). Discussion on methods of soil dehydrogenase determination. Int. J. Environ. Sci. Technol..

[73] Eivazi F., Tabatabai M.A. (1977). Phosphatases in Soils. Soil Boil. Biochem..

[74] Li Y., Feng H., Chen J., Lu J., Wu W., Liu X., Li C., Dong Q., Siddique K.H.M. (2022). Biochar Incorporation Increases Winter Wheat (*Triticum aestivum* L.) Production with Significantly Improving Soil Enzyme Activities at Jointing Stage. Catena.

[75] Eivazi F., Tabatabai M.A. (1988). Glucosidases and Galactosidases in Soils. Soil Biol. Biochem..

