# Supplementary material for: Combined Application of Biochar and Plant Growth-Promoting Rhizobacteria Improves Heavy Metal and Drought Stress Tolerance in Zea mays
Source: Plants (Basel). 2024 Apr 19;13(8):1143. doi: 10.3390/plants13081143 (PMC11053748; doi:10.3390/plants13081143)
Supplement: Supplementary file 1 [file plants-13-01143-s001.zip › plants-2967051-supplementary.pdf]

Supplementary

Table S1. Physico-chemical characteristics of Groundnut shell biochar

Parameters	GS-BC
pH	7.77
Conductivity (mS cm^{-1})	2.61
CEC (Cmol Kg^{-1})	7.9
Moisture Content (%)	2.73 ± 1.93
Organic Matter (%)	95 ± 0.34
Volatile matter (%)	39.1 ± 0.1
Ash (%)	9.3 ±0.1
Yield %	43 ± 1.7
Surface area (m^2/gm)	3.54
Elemental Compostion(%)	
C	60.3
N	2.1
H	3.3
O	21.5
Nutrient contents (mg kg-1)	
P	1489.81 ± 4.93
K	17145.41 ± 21.38
Na	195.30 ± 0.71
Mg	1224.72 ± 3.21
Al	930.7 ± 5.23
Cu	0.51 ± 0.06
Fe	1053 ± 2.51
Mn	52.07 ± 0.13
Zn	20.71 ± 0.11
Cd	BDL
Cr	BDL
Ni	1.53 ± 0.17
As	1.10 ± 0.02

Values are means ± standard deviations of three samples. BDL- below detection limits.

Table S2. Heavy metal stress tolerance and plant growth-promoting features of ARN7.

**Characteristics**	**ARN7**					
Metal tolerance level (mg L^{-1})						
Ni	200					
Cu	100					
Zn	400					
Cr	200					
Cd	100					
In vitro plant growth promotion						
Shoot length (cm)	2.97 ± 0.25					
Root length (cm)	14.23 ± 0.25					
Fresh weight (g $plant^{-1}$)	0.024 ± 0.001					
**PGP traits**	**Treatment**					
	Control	PEG 15%	PEG 30%	HM	PEG 15% + HM	PEG 30% + HM
IAA production (µg mL^{-1})	2.6 ± 0.19	2.3 ± 0.08	2.4 ± 0.08	1.9 ± 0.04	1.9 ± 0.07	2.0 ± 0.15
Siderophore production (%)	13.8 ± 1.78	14.6 ± 0.60	5.2 ± 1.03	10.9 ± 0.59	6.8 ± 2.16	3.2 ± 0.87
Catechol (µg mL^{-1})	-	-	-	-	-	-
Hydroxamate (µg mL^{-1})	41.0 ± 1.01	37.0 ± 2.13	27.0 ± 1.04	12.7 ± 2.06	10.0 ± 1.21	5.0 ± 0.09
P solubilization (mg mL^{-1})	90.9 ± 3.83	58.3 ± 1.49	29.7 ± 5.95	80.6 ± 3.25	47.6 ± 4.36	25.0 ± 2.99
Exopolysaccharides production (mg mL^{-1})	2.1 ± 0.01	4.0 ± 0.01	4.4 ± 0.03	4.3 ± 0.04	7.8 ± 0.05	9.1 ± 0.05

HM – heavy metal (Nickel -150 mg L^{-1}+ Zinc 300 mg L^{-1}); PEG – Polyethylene Glycol 6000; Values are means ± standard deviations of three samples.

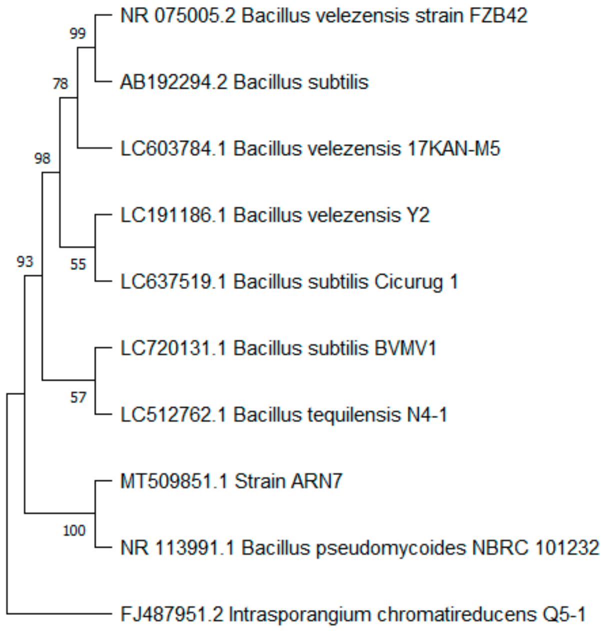

Figure S1. Phylogenetic tree showing the relationship of partial 16S rRNA gene sequences of ARN7 with other related sequences obtained from NCBI database. The tree was clustered with the neighbor-joining method using MEGA 11 package

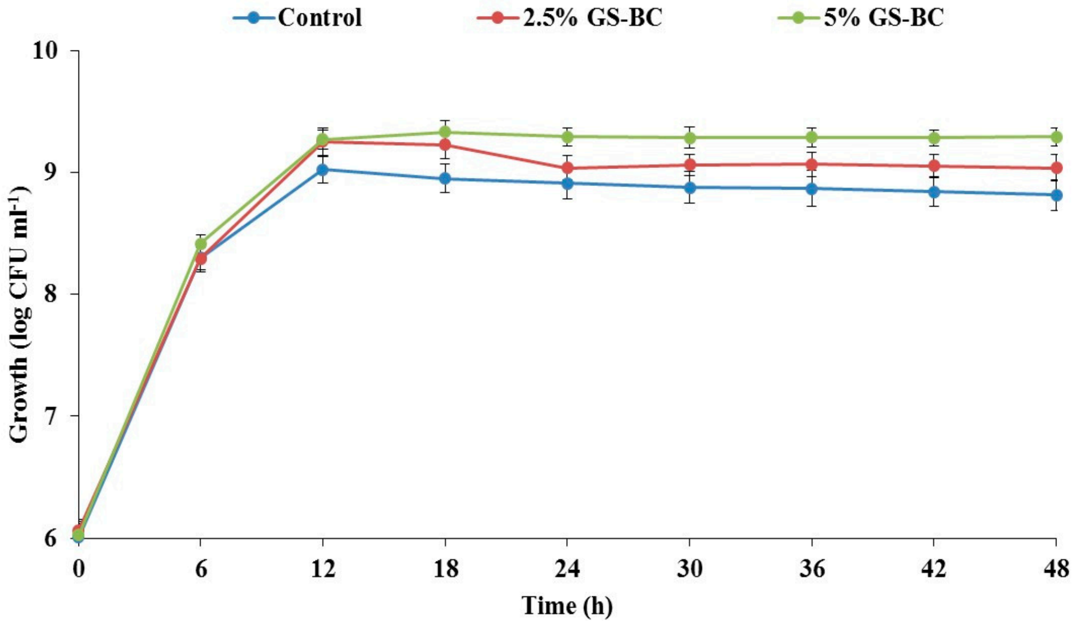

Figure S2. Growth pattern of ARN7 in tryptone soy broth medium supplemented with 0, 2.5 or 5% biochar.
